# Vector-borne pathogen evaluation and phylogenetic analysis in working K9 dogs in Iraq: implications for canine and public health

**DOI:** 10.1007/s00436-026-08723-0

**Published:** 2026-07-06

**Authors:** Mustafa Alsarraf, Christina Kuchler, Mohammed Alsarraf, Anna Bajer, Dagmara Wężyk, Barbara Eigner, Sadeer Abdul Ameer Hamed, Hans-Peter Fuehrer

**Affiliations:** 1 Department of Microbiology, Molecular Genetics and Genomics, Centre for Advanced Materials and Technologies, CEZAMAT, Warsaw University of Technology, 19 Poleczki St, Warsaw, 02-822 Poland; 2 Institute of Parasitology, Department of Biological Sciences and Pathobiology, University of Veterinary Medicine, Veterinaerplatz 1, Vienna, 1210 Austria; 3 Department of Eco-epidemiology of Parasitic Diseases, Institute of Developmental Biology and Biomedical Sciences, Faculty of Biology, University of Warsaw, Miecznikowa 1, Warsaw, 02-096 Poland; 4 Small Animal Unit, Adan Veterinary Hospital, Adan Square, 10011 Baghdad, Iraq

**Keywords:** K9, *Babesia* sp., *Mycoplasma haemocanis*, Iraq

## Abstract

Canine (K9) units are essential in law enforcement and military operations; they are often at higher risk of vector-borne infections due to their working and kennel environments. This study aimed to investigate the prevalence of vector-borne pathogens, including *Babesia* sp. and *Mycoplasma* spp., in military/police K9 dogs which visited the Adan veterinary hospital in Baghdad/Iraq in 2016–2017. 61 blood samples were collected on Whatman FTA cards and sent to the University of Veterinary Medicine in Vienna, Austria where they were tested with specific PCRs to get a better understanding about epidemiological situation of these vector-borne pathogens in K9s in Iraq. Two dogs tested positive, one with *Babesia* sp. and one with *Mycoplasma haemocanis*, making the prevalence 1.6% (1/61; 95% CI: 0.04–8.8%) for each parasite, the isolates were sequenced, and phylogenetic analysis was done to confirm their lineage with other sequences from the GenBank. This is the first study on vector-borne pathogens in K9s in Iraq and the first molecular record of infection with *M. haemocanis* in K9s.

**Supplementary Information:**

The online version contains supplementary material available at https://doi.org/10.1007/s00436-026-08723-0.

## Highlights

First epidemiological study on vector-borne pathogens in K9s in Iraq.

First record of *M. haemocanis* in K9s in Iraq.

The prevalence of these vector-borne pathogens is possibly low due to good living conditions of these dogs.

**Supplementary Information:**

The online version contains supplementary material available at https://doi.org/10.1007/s00436-026-08723-0.

## Introduction

Canine units, commonly referred to as K9 units, have been integral to law enforcement and military operations for centuries. In modern times, breeds such as German Shepherds, Belgian Malinois and Labrador Retrievers are preferred for their intelligence, agility, and olfactory capabilities. Military working dogs are at greater risk of vector-borne infections because of their kennel environments and regular contact with wildlife and vectors(Meyers et al. 2021).

The prevalence of the Apicomplexan *Babesia* sp. was previously documented in 5.1% (16/310) of pet dogs in Iraq including dogs of different breeds (Badawi and Yousif 2020), in another study based on 97 stray dogs no infection with *Babesia* sp. was found (Otranto et al. 2019). Hemotropic Mycoplasma were previously studied in dogs in Iraq and revealed a 3.8% prevalence (6/155) with *Mycoplasma haematoparvum* but no records of *Mycoplasma haemocanis* are to date in Iraq (Saeed and Badawi 2024). Interestingly, prevalence of *M. haemocanis* was 15.7% and 17.8% respectively in neighbouring countries like Saudi Arabia and Iran (Alanazi et al. 2020; Beus et al. 2024).

Baghdad is characterized by a hot desert climate with long, dry summers and mild winters, conditions that support the activity of several arthropod vectors, particularly ticks, responsible for transmitting canine vector-borne pathogens. The studied K9 dogs were housed in dedicated military and police kennels and underwent regular veterinary care and prophylactic ectoparasite control. The aim of this study was to examine an infection and prevalence of vector-borne pathogens in K9 police/military dogs which visited the Adan Veterinary Hospital in Baghdad/Iraq in 2016–2017. We hypothesized that these dogs are prone to infection with a variety of vector-borne pathogens due to their working and housing conditions, and the general arid climate of Iraq which has an abundance of parasitic vectors that thrive in it like mosquitoes, ticks etc.

## Materials and methods

### Sample collection

During this study, blood samples were taken from 61 dogs of different age groups at the Adan Veterinary Hospital in Baghdad between 2016 and 2017 during routine health check-ups where general physical examination is conducted, X-ray imaging for the heart and lungs, ultrasonography for the abdomen and a complete blood count (CBC) is done at the lab for each dog, dogs are generally checked every 3 months. The sampling included 25 females, 34 males and two individuals of unknown sex. These dogs belong to the category of K9 dogs, also known as police or military dogs. They are used for various tasks, including the search for drugs, explosives and counterterrorism activities. Most dogs stationed in Baghdad while four dogs stationed in Al Diwaniyah, one in Al-Kut, one in Hilla and one in Salah Aldin while three dogs were part of the mobile units moving between different regions of the country. The dogs were of various breeds, the majority being German Shepherds and Belgian Malinois.

Blood samples were collected on Whatman FTA cards (Kent, United Kingdom), labelled and stored in sealed bags at room temperature. Basic data on each dog, age, sex, breed and health condition was also recorded.

After collection, the samples were transferred to the University of Veterinary Medicine Vienna and for further laboratory processing.

### DNA extraction

Pieces of 4 × 4 mm size of FTA card were soaked in 100 µl phosphate-buffered saline solution (PBS pH 7.2–7.4) and stored overnight at the temperature of + 4 °C. The DNA extraction was done according to the technique previously described by Fuehrer et al. (2010). After the extraction of the DNA using the special InstaGene matrix, the tubes were stored at -20 °C until further analysis.

### Molecular detection

Different vector-borne pathogens were tested including *Mycoplasma* spp. and *Babesia* sp., *Anaplasma* spp., Rickettsia, Filarioidea, *Bartonella* spp., *Hepatozoon* spp., *Theileria* spp., and *Trypanosoma* spp. using species specific genes (Norman et al. 1995; Jensen et al. 2000; Parola et al. 2000; Criado-Fornelio et al. 2003; Vitorino et al. 2003; Zintl et al. 2011; Hamer et al. 2013; Hodžić et al. 2015; Peña-Espinoza et al. 2023).

In this study we used conventional and nested PCRs. PCR was performed in a total reaction volume of 20 µL using 5× Green Reaction Buffer, GoTaq^®^ Polymerase, and two specific primers. The reactions were run on an Eppendorf Mastercycler^®^. PCR products were analysed by electrophoresis on 1.8% agarose gels stained with Midori Green Advance^®^. Gels were run for approximately 45 min at 120 V, and DNA bands were visualized under UV light using the ChemiBIS genXpress system. Positive samples were subsequently selected for further analysis.

All amplicons were sent for Sanger sequencing at a commercial company Microsynth Austria GmbH (Vienna, Austria). Obtained sequences were aligned, trimmed and edited using CodonCode Aligner 10.0.2 (CodonCode Corp., Centerville, MA, USA).

### Phylogenetic analysis

Sequences were aligned using ClustalW (MEGA 12) with sequences obtained from the GenBank. Phylogenetic trees were computed using maximum likelihood method for 18 S rRNA sequences of *Babesia* spp., and the 16 S rRNA sequences of *Mycoplasma* spp. The evolutionary Tamura model (Tamura 1992)was chosen based on the model test by Mega 12 (Kumar et al. 2024) and bootstrapped over 1000 randomly generated sample trees that have invariants sites. Obtained sequences were accompanied by reference sequences from the GenBank database and relevant outgroups were included in the analysis as well.

## Results

Out of the 61 tested dogs, one dog (young male Belgian Malinois) tested positive for *Babesia* sp., while one young male Doberman tested positive for *M. haemocanis*. Thus, each parasite had a prevalence of 1.6% (1/61; 95% CI: 0.04–8.8%), and the combined prevalence of both infections was 3.28% (2/61; 95% CI: 0.40-11.35%).

The 374 bp-long *Babesia* sp. (PV631134) sequence showed rather low similarity of 97.3% to *Babesia canis canis* sequence from a dog from Poland (KT844889), while the 553 bp-long *Mycoplasma haemocanis* (PV640428) sequence showed a 100% similarity to *M. haemocanis* from a dog from Iraq (PP944853).

**Fig. 1:**
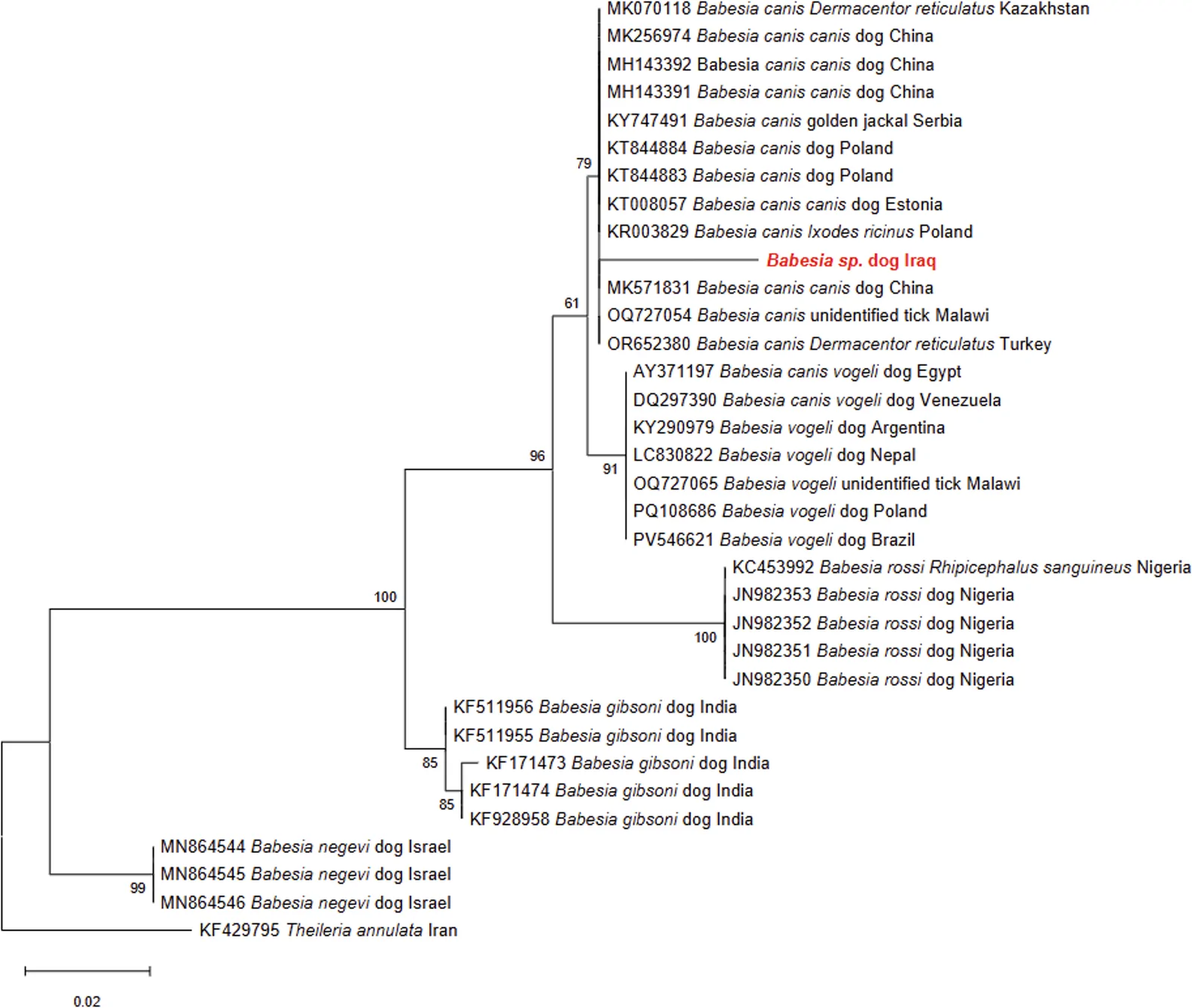
The phylogeny was inferred using the Maximum Likelihood method and Tamura (1992) model of nucleotide substitutions and the tree with the highest log likelihood (-919.36) is shown. The percentage of replicate trees in which the associated taxa clustered together (1,000 replicates) is shown next to the branches based on the 18 S ribosomal RNA gene

The phylogenetic analysis has shown that *Babesia* sp. sequence from this study formed a separate branch within *Babesia canis canis* clade (Fig. 1).

**Fig. 2:**
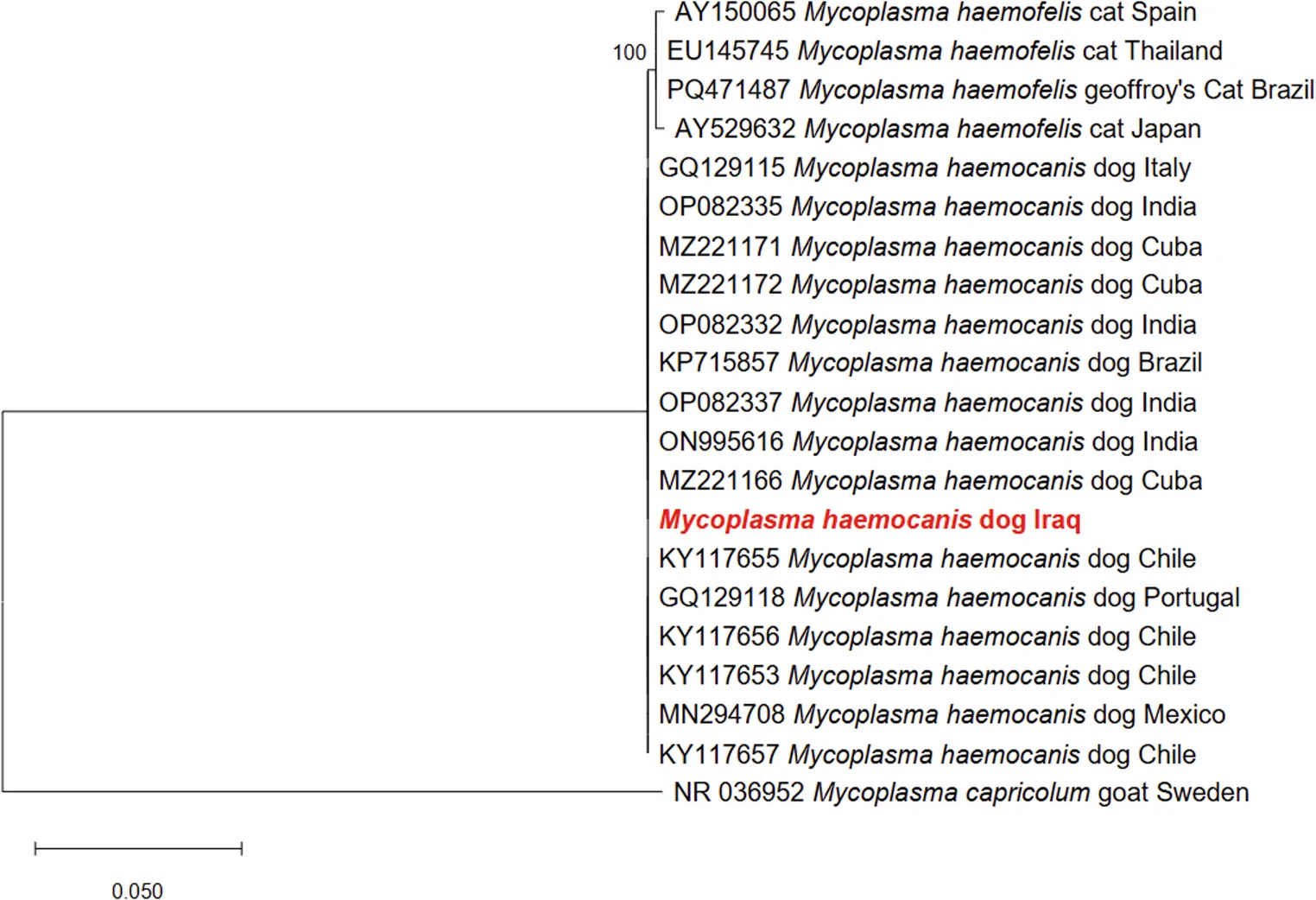
The phylogeny was inferred using the Maximum Likelihood method and Kimura (1980) 2-parameter model of nucleotide substitutions and the tree with the highest log likelihood (-1,271.33) is shown. The percentage of replicate trees in which the associated taxa clustered together (1,000 replicates) is shown next to the branches based on the 16 S ribosomal RNA gene

The *M. haemocanis* sequence from this study grouped together with other *M. haemocanis* sequences from dogs from different countries worldwide (Fig. 2).

All sequences were submitted to the GenBank, and the following Accession numbers were obtained: PV631134 for *Babesia* sp. and PV640428 for *Mycoplasma haemocanis*.

## Discussion

Identification of *Babesia* sp. and *M. haemocanis* in this study constitutes the first records of infection by these vector-borne pathogens in K9s in Iraq. When comparing the overall parasite prevalence found in this study (1.6%) with previous research focused specifically on *Babesia canis* in pet dogs, the earlier work by Badawi and Yousif (2020) reported a considerably higher prevalence of 5.1%; it is important to note that K9s are considered public property and they undergo weekly veterinarian check-ups, extensive care for their hygiene and sheltering (due to their cost and the cost of their training), plus regular prophylactic treatments against arthropod vectors, all these factors are less commonly practiced in pet dogs and non-existent in stray dogs. Previous studies have shown that all these factors ensure that the service dogs operate at optimum capacity and conduct their field work thoroughly (Cobb et al. 2021).

The *Mycoplasma haemocanis* sequences obtained in this study showed 100% identity with previously published sequences in GenBank. This indicates that the detected strain corresponds to the commonly reported *M. haemocanis* circulating globally.

In contrast, the *Babesia* sequences displayed several single nucleotide polymorphisms (SNPs) when compared with available *B. canis* sequences. Because molecular data from Iraq are extremely limited, it remains uncertain whether these variants represent a divergent strain of *B. canis*, a lineage unique to Iraq, or potentially a previously uncharacterized species, This observation is based on the fact that the sequence from Iraq clusters within the *Babesia canis* branch while showing divergence from other *Babesia* species such as *B. gibsoni* and *B. negevi*.

Although *Babesia canis* and *Mycoplasma haemocanis* are not considered major zoonotic pathogens, they share arthropod vectors with several pathogens of public health importance. Therefore, surveillance of vector-borne pathogens in working dogs may contribute to broader monitoring efforts within a One Health framework. This study has several limitations. The relatively small sample size (*n* = 61) and low number of PCR positive dogs resulted in wide 95% confidence intervals and precluded meaningful analysis of potential risk factors. Clinical and hematological data for the positive dogs were unavailable, preventing correlation between molecular findings and clinical status. In addition, ticks and other ectoparasites were not collected, limiting assessment of local vector infection and transmission dynamics. Sampling was restricted to 2016–2017, precluding evaluation of seasonal trends, and the inclusion of dogs undergoing routine veterinary examinations may have introduced bias toward healthier animals receiving regular prophylactic care. Future studies should incorporate larger sample sizes, longitudinal sampling, and integrated dog-vector surveillance. Routine ectoparasite control, veterinary monitoring, and molecular screening prior to deployment may further support the health of working K9 dogs in endemic regions.

## Conclusions

This is the first study on vector-borne pathogens in police and military dogs in Iraq and the first molecular record of infection with *Babesia* sp. and *M. haemocanis* in these K9s. It also confirms that advanced care of the dogs with proper hygiene, regular veterinarian check-ups and regular prophylactic treatment can significantly lower the rate of infection with these vector-borne pathogens when compared to the higher prevalence observed in pet and stray dogs. It is recommended that more studies are conducted on K9s, and more studies are to be conducted on dogs in general in Iraq to get better understanding of the epidemiological situation of these vector-borne pathogens.

## Supplementary Information

Below is the link to the electronic supplementary material.

Supplementary Material 1 (DOCX 57.7 KB)

## Data Availability

Sequences were uploaded to NCBI GenBank.
