# Supplementary material for: Vector-borne pathogen evaluation and phylogenetic analysis in working K9 dogs in Iraq: implications for canine and public health
Source: Parasitol Res. 2026 Jul 6;125(1):116. doi: 10.1007/s00436-026-08723-0 (PMC13616852; doi:10.1007/s00436-026-08723-0)
Supplement: Supplementary file 1 [file 436_2026_8723_MOESM1_ESM.docx]

**Parasite Evaluation and Phylogenetic Analysis in Working K9 Dogs in Iraq: Implications for Canine and Public Health**

**Mustafa Alsarraf^1†^, Christina Kuchler^2†^, Mohammed Alsarraf^3^, Anna Bajer^3^, Dagmara Wężyk^3^, Barbara Eigner^2^, Sadeer Abdul Ameer Hamed^4^, Hans-Peter Fuehrer^2^**

1. Department of Microbiology, Molecular Genetics and Genomics, Centre for Advanced Materials and Technologies, CEZAMAT, 19 Poleczki St, Warsaw 02-822, Poland.
2. Parasitology, Department of Biological Sciences and Pathobiology, University of Veterinary Medicine, Veterinaerplatz 1, 1210 Vienna, Austria.
3. Department of Eco-epidemiology of Parasitic Diseases, Institute of Developmental Biology and Biomedical Sciences, Faculty of Biology, University of Warsaw, Warsaw, Poland, Miecznikowa 1, 02-096 Warsaw, Poland
4. Adan Veterinary Hospital, Small Animal Unit, Adan Square, 10011, Baghdad, Iraq

†: These authors contributed equally to the work (Co-First authors).

Corresponding author: Hans-Peter Fuehrer, Email: [Hans-Peter.Fuehrer@vetmeduni.ac.at](mailto:Hans-Peter.Fuehrer@vetmeduni.ac.at)

**Journal Name: Parasitology Research**

**S1** Established PCR protocols

| **Species / Group** | **Target Gene** | **Primer Name** | **Sequence (5′→3′)** | **Annealing Temperature** | **Reference*** |
| --- | --- | --- | --- | --- | --- |
| ***Mycoplasma* spp.** | 16S rRNA | HBT-F | ATA CGG CCC ATA TTC CTA CG | 60 °C | (Criado-Fornelio et al. 2003) |
|  |  | HBT-R | TGC TCC ACC ACT TGT TCA | 60 °C |  |
| **Piroplasmida** | 18S rRNA (nested PCR) | BTH-1F | CCT GAG AAA CGG CTA CCA CAT CT | 68 °C | (Zintl et al. 2011) |
|  |  | BTH-1R | TTG CGA CCA TAC TCC CCC CA | 68 °C |  |
|  | 18S rRNA (nested PCR) | GF2 | GTC TTG TAA TTG GAA TGA TGG | 60 °C |  |
|  |  | GR2 | CCA AAG ACT TTG ATT TCT CTC | 60 °C |  |
| **Trypanosomatida** | 18S rRNA (nested PCR) | Tryp_18S_F1 | GTG GAC TGC CAT GGC GTT GA | 56 °C | (Peña-Espinoza et al. 2023) |
|  |  | Tryp_18S_R1 | CAG CTT GGA TCT CGT CCG TTG A | 56 °C |  |
|  | 18S rRNA (nested PCR) | Tryp_18S_F2 | CGA TGA GGC AGC GAA AAG AAA TAG AG | 56 °C |  |
|  |  | Tryp_18S_R2 | GAC TGT AAC CTC AAA GCT TTC GCG | 56 °C |  |
| **Filarioidea** | COI (Touchdown PCR) | COIint-F | TGA TTG GTG GTT TTG GTA A | 51–47 °C → 45 °C | (Hamer et al. 2013) |
|  |  | COIint-R | ATA AGT ACG AGT ATC AAT ATC | 51–47 °C → 45 °C |  |
| **Anaplasmataceae** | 16S rRNA | EHR16SD_for | GGT ACC YAC AGA AGA AGT CC | 54 °C | (Parola et al. 2000) |
|  |  | EHR16SR_rev | TAG CAC TCA TCG TTT ACA GC | 54 °C |  |
| ***Rickettsia* spp.** | 23S/5S rRNA | ITS-F | GAT AGG TCG GGT GTG GAA G | 52 °C | (VITORINO et al. 2003) |
|  |  | ITS-R | TCG GGA TGG GAT CGT GTG | 52 °C |  |
| ***Bartonella* spp.** | 16S–23S rRNA | bartgd_for | GAT GAT GAT CCC AAG CCT TC | 60 °C | (Jensen et al. 2000) |
|  |  | B1623_rev | AAC CAA CTG AGC TAC AAG CC | 60 °C |  |
|  | gltA | BhCS.781p | GGG GAC CAG CTC ATG GTG G | 54 °C | (Norman et al. 1995) |
|  |  | BhCS.1137n | AAT GCA AAA AGA ACA GTA AAC A | 54 °C |  |
| ***Hepatozoon* spp.** | 18S rRNA | H14Hepa18SFw | GAA ATA ACA ATA CAA GGC AGT TAA AAT GCT | 58 °C | (Hodžić et al. 2015) |
|  |  | H14Hepa18SRv | GTG CTG AAG GAG TCG TTT ATA AAG A | 58 °C |  |

* References correspond to the main manuscript reference list
